# Datasets related to in‐land water for limnology and remote sensing applications: distance‐to‐land, distance‐to‐water, water‐body identifier and lake‐centre co‐ordinates

**DOI:** 10.1002/gdj3.32

**Published:** 2016-02-19

**Authors:** Laura Carrea, Owen Embury, Christopher J. Merchant

**Affiliations:** ^1^ Department of MeteorologyUniversity of ReadingReadingUK

**Keywords:** limnology, global, lake, satellite, surface water temperature

## Abstract

Datasets containing information to locate and identify water bodies have been generated from data locating static‐water‐bodies with resolution of about 300 m (1/360^∘^) recently released by the Land Cover Climate Change Initiative (LC CCI) of the European Space Agency. The LC CCI water‐bodies dataset has been obtained from multi‐temporal metrics based on time series of the backscattered intensity recorded by ASAR on Envisat between 2005 and 2010. The new derived datasets provide coherently: distance to land, distance to water, water‐body identifiers and lake‐centre locations. The water‐body identifier dataset locates the water bodies assigning the identifiers of the Global Lakes and Wetlands Database (GLWD), and lake centres are defined for in‐land waters for which GLWD IDs were determined. The new datasets therefore link recent lake/reservoir/wetlands extent to the GLWD, together with a set of coordinates which locates unambiguously the water bodies in the database. Information on distance‐to‐land for each water cell and the distance‐to‐water for each land cell has many potential applications in remote sensing, where the applicability of geophysical retrieval algorithms may be affected by the presence of water or land within a satellite field of view (image pixel). During the generation and validation of the datasets some limitations of the GLWD database and of the LC CCI water‐bodies mask have been found. Some examples of the inaccuracies/limitations are presented and discussed. Temporal change in water‐body extent is common. Future versions of the LC CCI dataset are planned to represent temporal variation, and this will permit these derived datasets to be updated.

## Dataset

Identifier: 10.5285/6be871bc‐9572‐4345‐bb9a‐2c42d9d85ceb

Creator: L. Carrea, O. Embury and C.J. Merchant

Title: Distance to land dataset

Publisher: NERC Centre for Environmental Data Analysis (CEDA)

Publication year: 2015

Resource type: Dataset

Version: 1.0

Identifier: 10.5285/6be871bc‐9572‐4345‐bb9a‐2c42d9d85ceb

Creator: L. Carrea, O. Embury and C.J. Merchant

Title: Distance to water dataset

Publisher: NERC Centre for Environmental Data Analysis (CEDA)

Publication year: 2015

Resource type: Dataset

Version: 1.0

Identifier: 10.5285/6be871bc‐9572‐4345‐bb9a‐2c42d9d85ceb

Creator: L. Carrea, O. Embury and C.J. Merchant

Title: Water bodies identifiers dataset

Publisher: NERC Centre for Environmental Data Analysis (CEDA)

Publication year: 2015

Resource type: Dataset

Version: 1.0

Identifier: 10.5285/6be871bc‐9572‐4345‐bb9a‐2c42d9d85ceb

Creator: L. Carrea, O. Embury and C.J. Merchant

Title: Lake centre and lake info dataset

Publisher: NERC Centre for Environmental Data Analysis (CEDA)

Publication year: 2015

Resource type: Dataset

Version: 1.0

## Introduction

A global map of open permanent water bodies at ∼300 m resolution was released in October 2014 by the Land Cover Climate Change Initiative (LC CCI) project (Defourny & Bontemps, 2012; Bontemps *et al*., 2015). This is of widespread interest, since water bodies play an important role in climate and global water cycles. A recent study (Verpoorter *et al*., 2014) calculated that there are globally 117 million lakes larger than 0.002 km^2^. Knowing the geographical location and distribution of inland water bodies is relevant to the understanding of regional environments, climate change, agricultural sustainability, present and future water resources (Wetzel, 2001) and many other subjects.

The datasets presented here have been derived from the LC CCI open permanent water‐bodies dataset (Santoro *et al*., 2015). There are four consistent global datasets: distance‐to‐land, distance‐to‐water, water‐body identifiers and water‐body centres (the latter two provided for the 3750 largest water bodies).

We derived these datasets as auxiliary information for measuring lake surface water temperature (LSWT) by thermal remote sensing (MacCallum & Merchant, 2012), within the project Globolakes (http://www.globolakes.ac.uk). However, the datasets are made available for their usefulness to a broader scientific community in different applications.

## 1 The water‐bodies dataset of the LC CCI

The new datasets are derived from global data on open, permanent water bodies (inland water and oceans) from the LC CCI project (http://cci.esa.int), version 1. LC CCI used observations from the Envisat Advanced Synthetic Aperture Radar (ASAR) combined with the Shuttle Radar Topography Mission (SRTM) Water Body data (SWBD) and data from the Medium‐spectral Resolution Imaging Spectrometer (MERIS). The land/water classification was derived from multi‐temporal metrics based on time series of the backscattered intensity recorded by the ASAR instrument between 2005 and 2010 (occasionally up to 2012 to avoid data voids). The main source of ASAR imagery is the Wide Swath Mode (WSM) at 150 m spatial resolution (Kirches *et al*., 2013).

**Figure 1:**
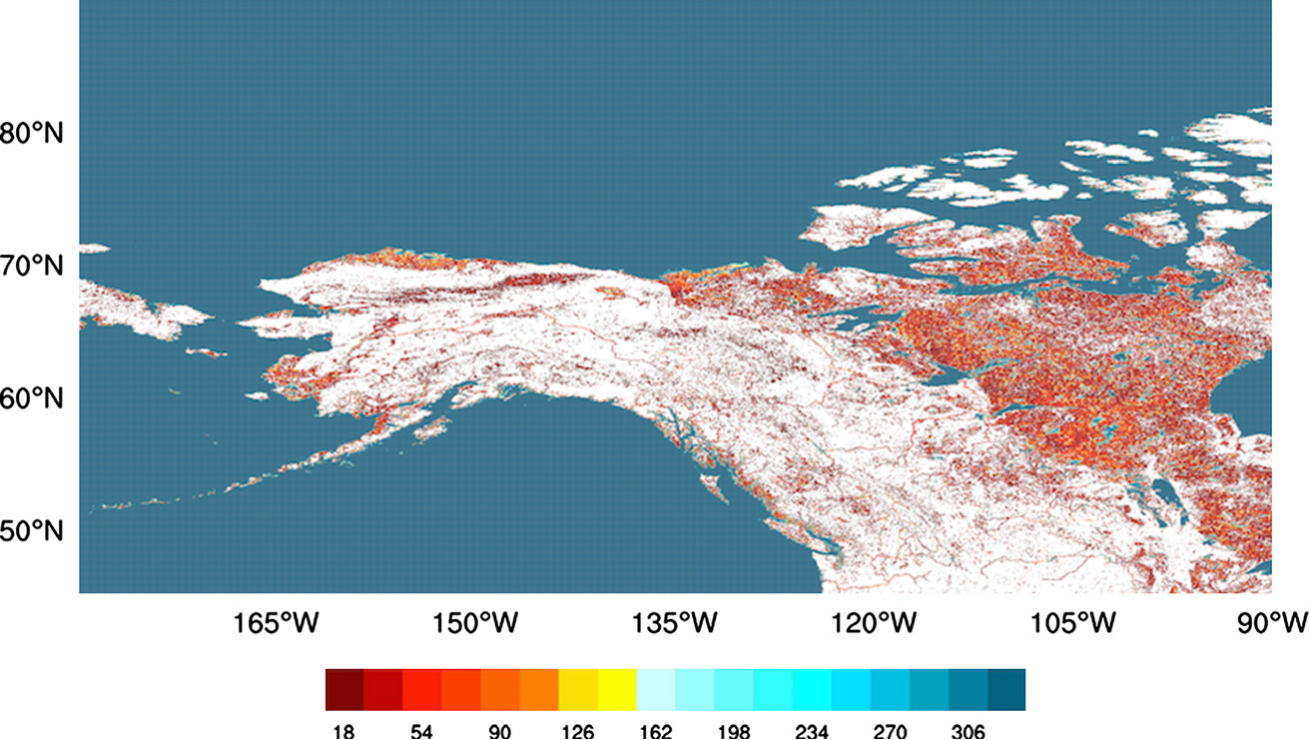
A portion of the global LC CCI water‐bodies map represented at 1/20^∘^ resolution. The colour shows the number of 1/360^∘^×1/360^∘^ cells identified as water per 1/20^∘^×1/20^∘^ latitude–longitude grid box, with a maximum value of 18×18 = 324.

**Figure 2:**
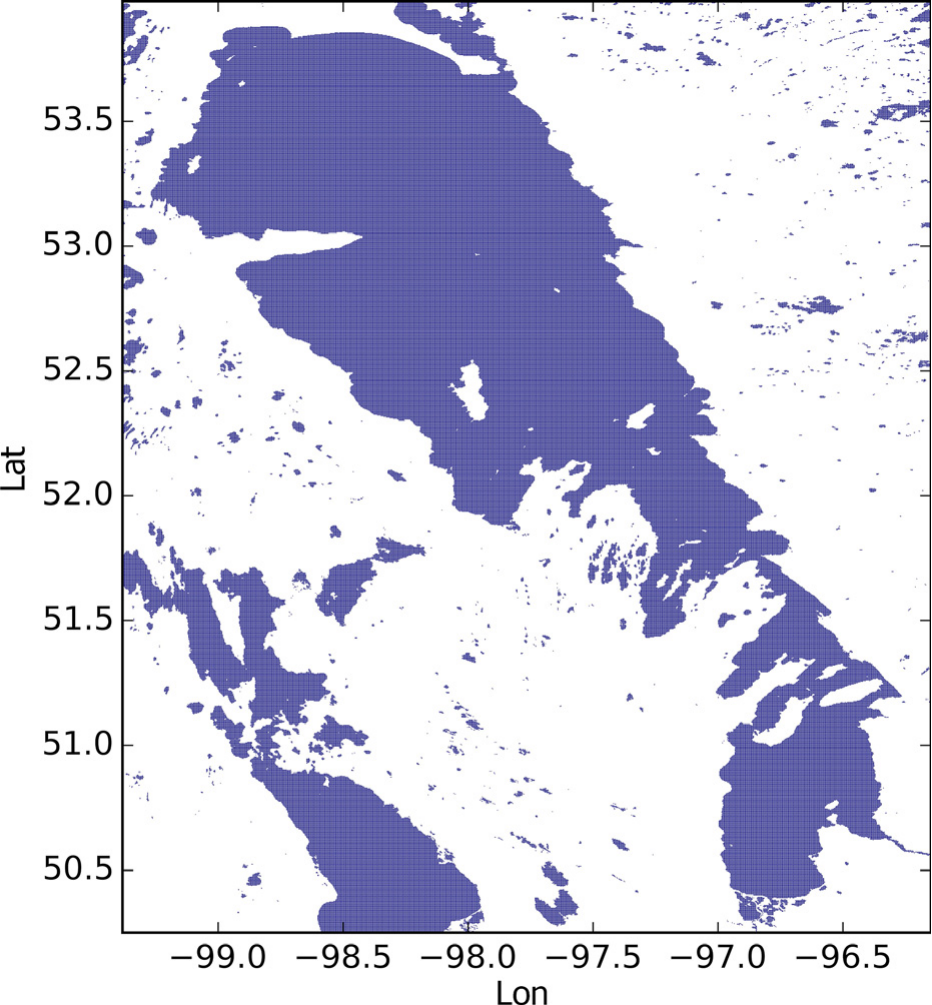
The area around Lake Winnipeg in Canada in the full resolution LC CCI water‐bodies dataset.

The map is at 1/360^∘^ resolution, which is about 300 m at the Equator and it is static. Figure 1 shows a portion of the map resampled at 1/20^∘^, where the color shows the number of 1/360^∘^×1/360^∘^ cells identified as water per 1/20^∘^×1/20^∘^ latitude–longitude grid box. Figure 2 shows the area around Lake Winnipeg in Canada at full resolution.

## 2 The distance‐to‐land dataset

Previous related datasets include: global calculations of distance to the nearest coast carried out at 1/25^∘^ resolution at the Goddard Space Flight Center (GSFC) for generating coastal ocean color products (available at http://www.ngdc.noaa.gov/) (Stumpf, 2012); calculations at very coarse global resolution for Point of Inaccessibility calculations (Garcia‐Castellanos & Lombardo, 2007).

**Figure 3:**
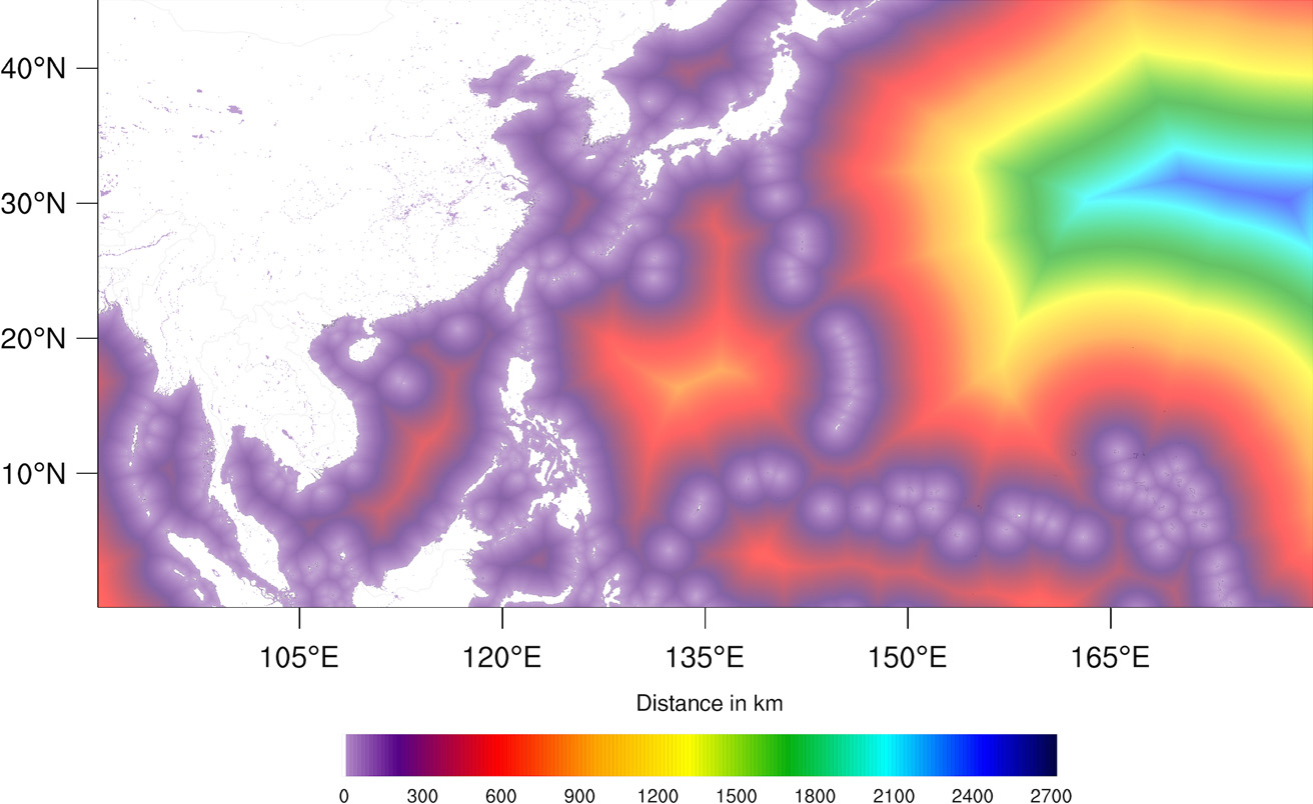
Extract of the distance‐to‐land dataset resampled at 1/20^∘^ for plotting. The colour scale relates to global distances.

**Figure 4:**
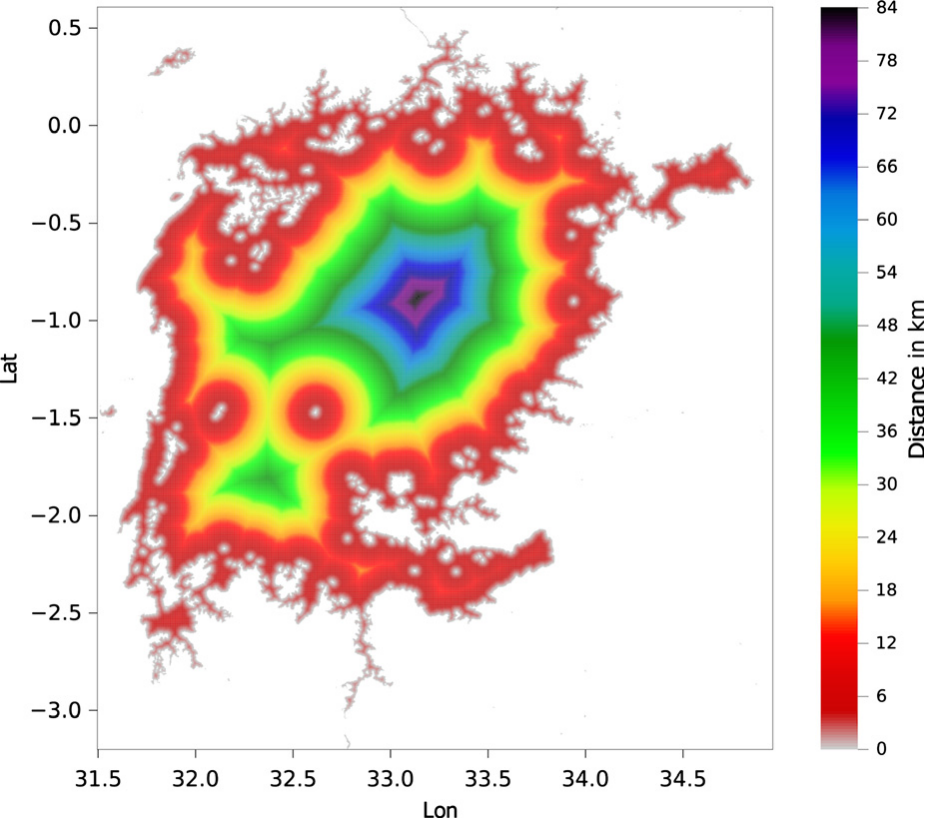
Lake Victoria in Tanzania: example of the distance‐to‐land dataset for inland water (plotted at full resolution).

The distance‐to‐land dataset presented here is calculated at finer resolution, and not only with reference to sea–land coastlines. It contains the great circle distance from the nearest land for each water cell of the water‐body dataset, for both sea and inland water. At 1/360^∘^ resolution, the LC CCI dataset contains 129 600×64 800∼8410^9^ cells and locating the nearest cell is computationally demanding. The algorithm to compute the great circle distance on Earth accurately is based on the haversine formula (Sinnot, 1984), and the accuracy of the calculation is well within 100 m:

(1) $$d=R\Delta \sigma =2R\arcsin\sqrt{\sin^{2}\Delta \frac{\lambda }{2}+\cos\lambda_{0}\cos\lambda_{1}\sin^{2}\frac{\Delta \phi }{2}}$$

where *R* is the Earth radius, Δ*σ* is the angle at the centre of the sphere between the two points, *λ*_0_ and *λ*_1_ are the latitudes of the two points, Δ*λ* is their difference and Δ*ϕ* is the difference of the longitudes of the two points. A latitudinally dependent Earth radius has been utilized:

(2) $$R\left(\lambda \right)=\sqrt{\frac{a^{2}\cos^{2}\lambda_{0}+b^{2}\cos^{2}\lambda_{1}}{a\cos^{2}\lambda_{0}+b\cos^{2}\lambda_{1}}}$$

where *a* = 6378.1370 km is the radius to the Equator and *b* = 6356.7523 km is the radius to the Poles. Figure 3 shows a portion of the distance‐to‐land dataset over a large scale, and Figure 4 shows an example of distance to land for inland water at full resolution.

**Figure 5:**
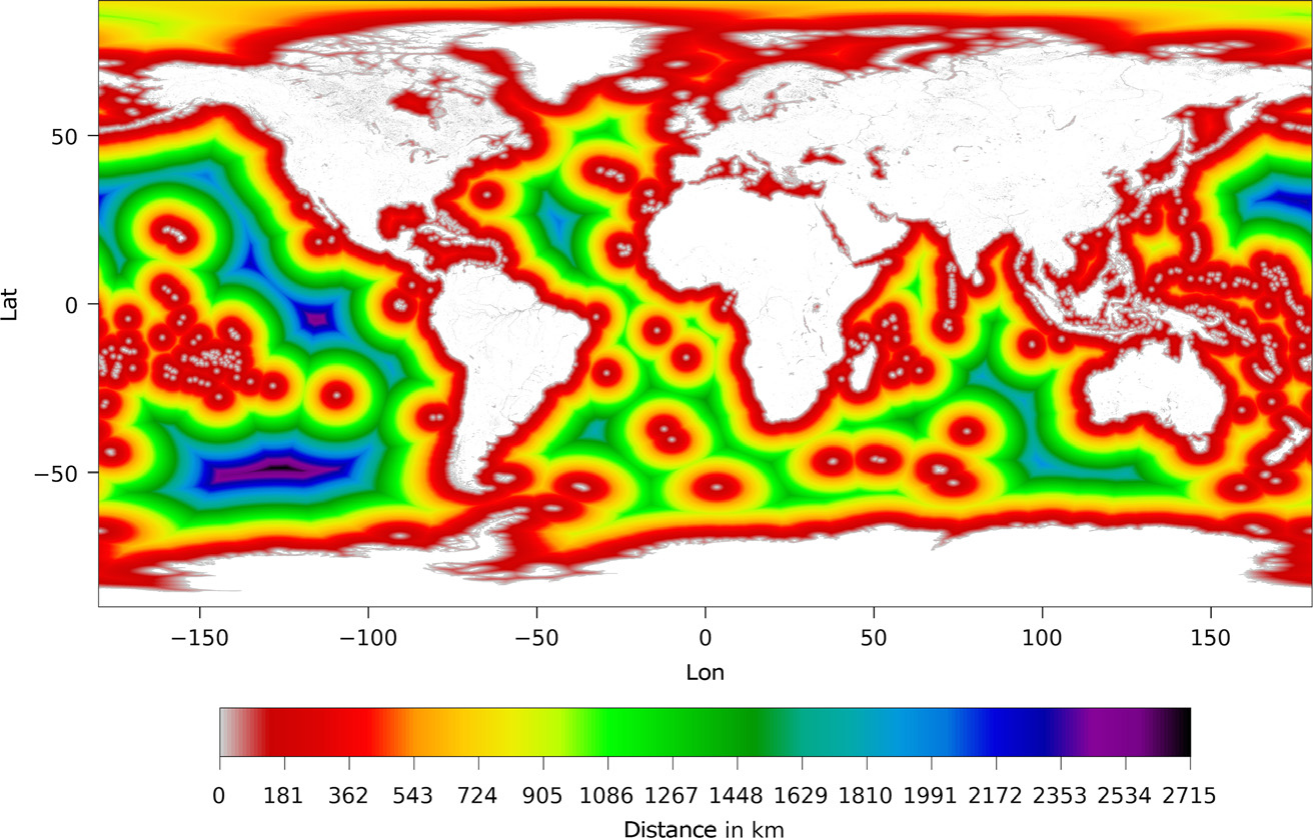
Global plot of the distance‐to‐land dataset at coarse resolution.

A global scale plot at coarse resolution is shown in Figure 5.

The validation of the distance‐to‐land dataset has been performed visually in comparison with the results of coarse‐resolution calculations from Garcia‐Castellanos & Lombardo 2007) and also by assessing values for extreme points. For example, we considered the most distant point from land, Point Nemo, the so‐called Pole of Inaccessibility of the Pacific Ocean. Point Nemo is located at *λ* = −48.87666 *ϕ* = −123.39333 at 2688 km equidistant from three small islands: Ducie Island (part of the Pitcairn Islands) in the north, Motu Nui (part of the Easter Islands) in the northeast, and Maher Island (near the larger Siple Island, off the coast of Marie Byrd Land, Antarctica) in the south. None of these three locations are present in the LC CCI mask. Near Motu Nui, the bigger Easter Island is present in the mask and near Maher Island the bigger Siple Island is present. Thus, the distance to the land of Point Nemo in the new dataset is slightly larger, 2691 km. This is acceptable for the remote sensing applications for which we have developed this dataset, such as checking the validity of a particular satellite observation for a land‐only or water‐only retrieval algorithm. There are further examples of small islands being absent from the underlying LC dataset in this version, such as most of the South Sandwich Islands or the Antipodes Island.

The distance‐to‐land values are organized in a Network Common Data Form (netCDF) file (for information on netCDF, see http://www.unidata.ucar.edu/software/).

## 3 The distance‐to‐water dataset

The position of water over land strongly influences the distribution of many species, including human populations. Kummu *et al*. 2011) reported an analysis of the relationship between inhabited places, distance to surface freshwater bodies, and climatic characteristics in different climate zones, showing that knowledge of the distance to water can be crucial for over 800 million people who still live without acceptable sources of drinking water (WHO‐UNICEF, 2010).

The distance‐to‐water dataset is similar in concept to that produced from the ‘GlobCover 2009’ dataset by Esri in 2014 (available at http://www.arcgis.com/home/item.html?id=46cbfa5ac94743e4933b6896f1dcecfd).

The distance‐to‐water presented here is complementary to the distance‐to‐water dataset described above, and it has been computed for each land pixel of the LC CCI product. The distance for each pixel is the distance to the nearest water cell. For the water cell the distance to water is 0 km. All water cells (inland and sea) are used, so the distance given is the distance to any water cell. Ice‐covered land areas are not treated as water, and by the nature of the input dataset, ephemeral surface water is not included. The furthest point from water in this sense is located in Antarctica.

**Figure 6:**
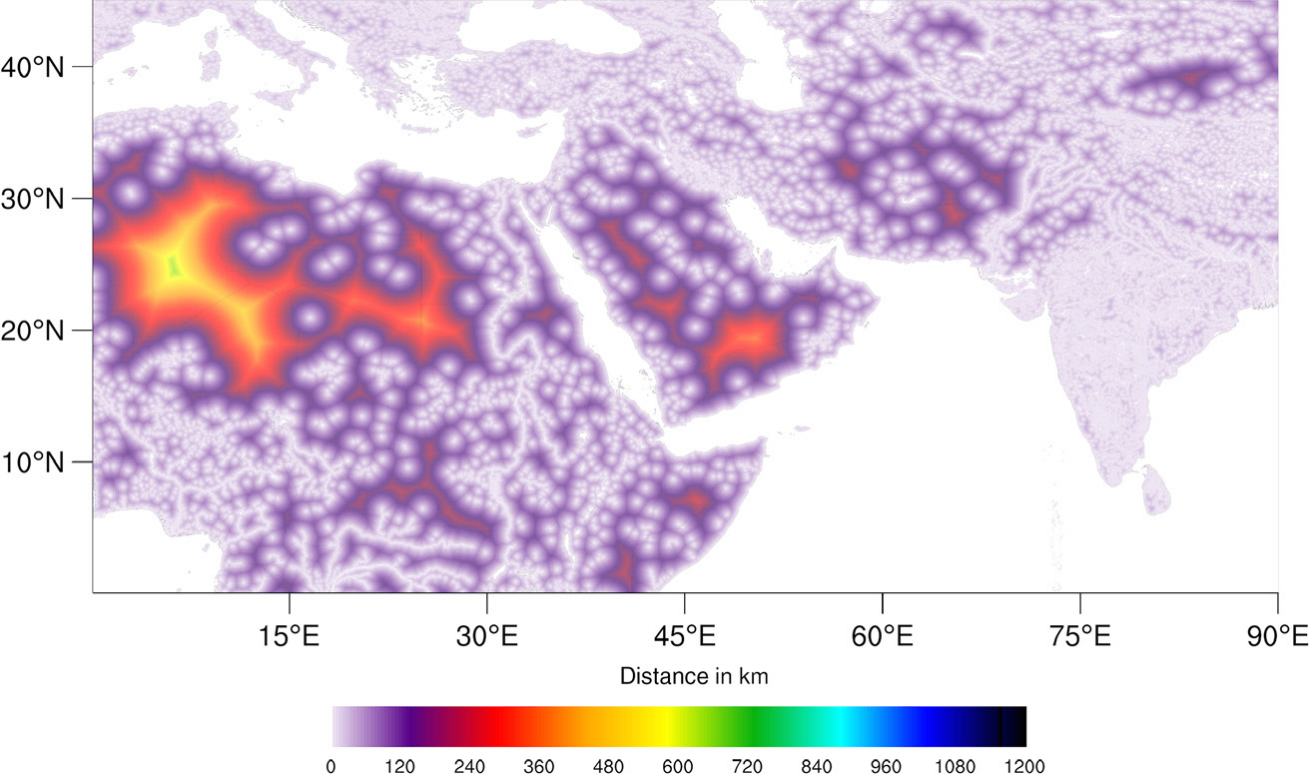
Extract of the distance‐to‐water dataset resampled at 1/20^∘^ for plotting. The colour scale relates to global distances.

**Figure 7:**
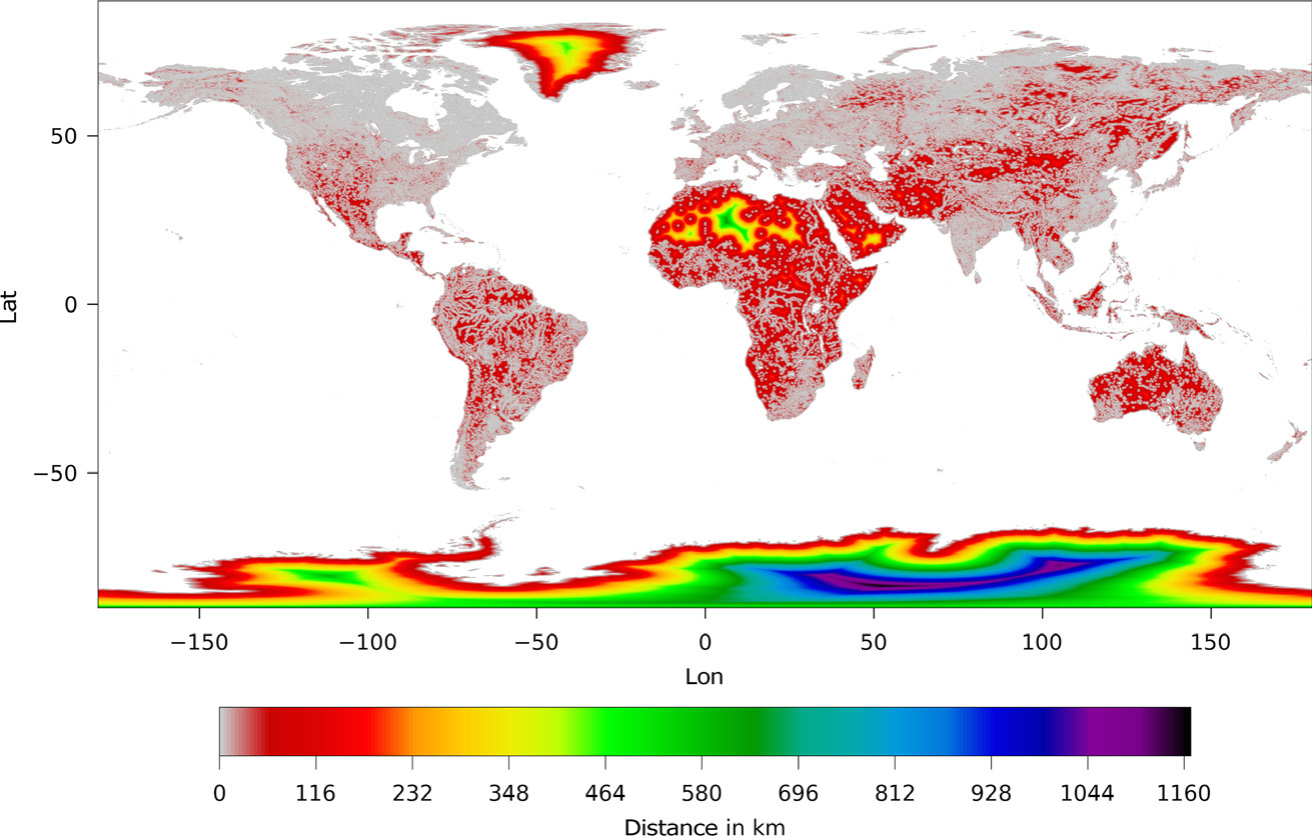
Global plot of the distance‐to‐water dataset at coarse resolution.

**Figure 8:**
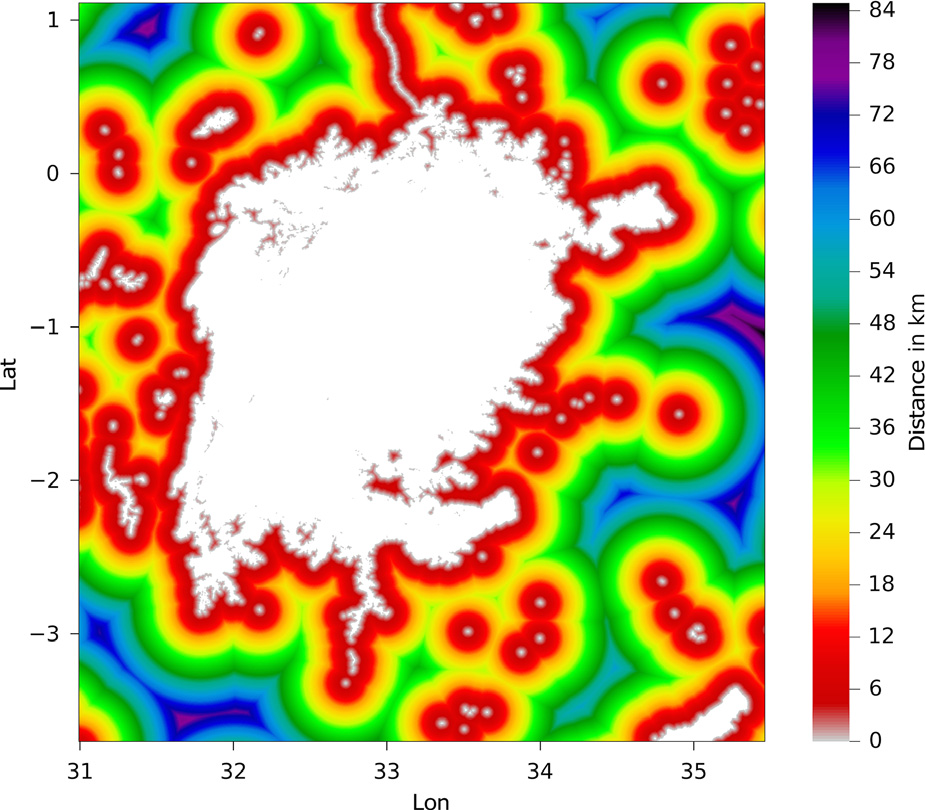
The area around Lake Victoria in Tanzania: extract of the distance‐to‐water dataset for inland water plotted at full resolution.

The method to generate the dataset is the same as for the distance to land. Figure 6 shows a portion of the distance‐to‐water dataset resampled at 1/20^∘^ for plotting. Figure 8 shows the correspondent area shown for the distance to land (see Figure 4) around Lake Victoria at full resolution. A global scale plot at coarse resolution is shown in Figure 7.

The distance‐to‐water values are also presented in a netCDF file.

## 4 Dataset of water‐body identifiers

The LC CCI water‐bodies mask only classifies pixels as water or land. For some applications, e.g., remote sensing of particular lakes, identifiers associated with a named water body are required. The dataset of water‐body labels contains a classification of the water pixels as belonging to sea, particular named lakes or reservoirs, and other inland water.

For inland water labeling, our dataset makes reference to the Global Lakes and Wetlands Database (GLWD) (Lehner & Döll, 2004). GLWD is organized in two main different databases:

- GLWD‐1: GLWD Level 1 which comprises the shorelines polygons of the 3067 largest lakes with area  > 50 km^2^ and the 654 largest reservoir of storage capacity  > 0.5 km^3^.
- GLWD‐2: GLWD Level 2 which includes a digital polygon global map of about 250 000 small lakes and reservoirs with area ≥ 0.1 km^2^ excluding the water bodies contained in GLWD‐1.

In some cases, a single GLWD lake identifier (ID) covers several lakes in a common basin, and sometimes it addresses a portion of a larger connected water body (perhaps one that is traditionally named as a distinct lake), as pointed out in Lehner & Döll 2004).

The dataset presented in this paper contains the classification of the water pixels into the following classes:

**‐1**: land
- **0**: sea
- from **1** to **3721**: all the GLWD‐1 water bodies
- 28 GLWD‐2 water bodies with IDs reported in Table 1
- **248614**: Curonian Lagoon between Lithuania and Russia
- **999999**: other water

**Table 1:** List of labels for the GLWD‐2 selected water bodies

Label	Name	Country	Type	Area (km^2^)
**4292**	BRATE	Romania	Lake	42.7
**4503**	MENDOTA	United States	Reservoir	40.6
**5506**	GREAT POND	United States	Reservoir	32.8
**6785**	ERKEN	Sweden	Lake	26.3
**6786**	TAY	United Kingdom	Lake	26.3
**7889**	MELVIN	Republic of Ireland; United Kingdom	Lake	22.5
**8089**	BADABAG	Romania	Lake	22.0
**9168**	DRANOV	Romania	Lake	19.3
**9322**	SUNAPEE	United States	Reservoir	19.0
**11740**	WINDERMERE	United Kingdom	Lake	15.1
**12262**	LEVEN	United Kingdom	Lake	14.4
**12471**	TROUT	United States	Lake	14.2
**12943**	KATRINE	United Kingdom	Lake	13.7
**13377**	DOUGLAS	United States	Lake	13.3
**13916**	GORGOVA	Romania	Lake	12.8
**15309**	ROSU	Romania	Lake	11.7
**16662**	JIJILA	Romania	Lake	10.8
**16814**	LUMINA	Romania	Lake	10.7
**17329**	MERHEI	Romania	Lake	10.4
**163748**	BASSENTHWAITE	United Kingdom	Lake	4.7
**164293**	ULLSWATER	United Kingdom	Lake	8.8
**164384**	DERWENT	United Kingdom	Lake	5.2
**208447**	MATITA	Romania	Lake	5.3
**208662**	FORTUNA	Romania	Lake	9.5
**208962**	ISAC	Romania	Lake	8.0
**209099**	PUIU	Romania	Lake	7.0
**211002**	AUBURN	United States	Reservoir	8.3
**213513**	SANABRIA	Spain	Lake	3.2

The water bodies area in Table 1 is as reported in GLWD. The additional lakes beyond GLWD‐1 were included for specific purposes related to the project under which this work is funded. GLWD‐2 is not addressed in its entirety because resources did not allow validation of the attribution of water cells to all GLWD‐2 water bodies. The IDs are organized in a netCDF file.

**Figure 9:**
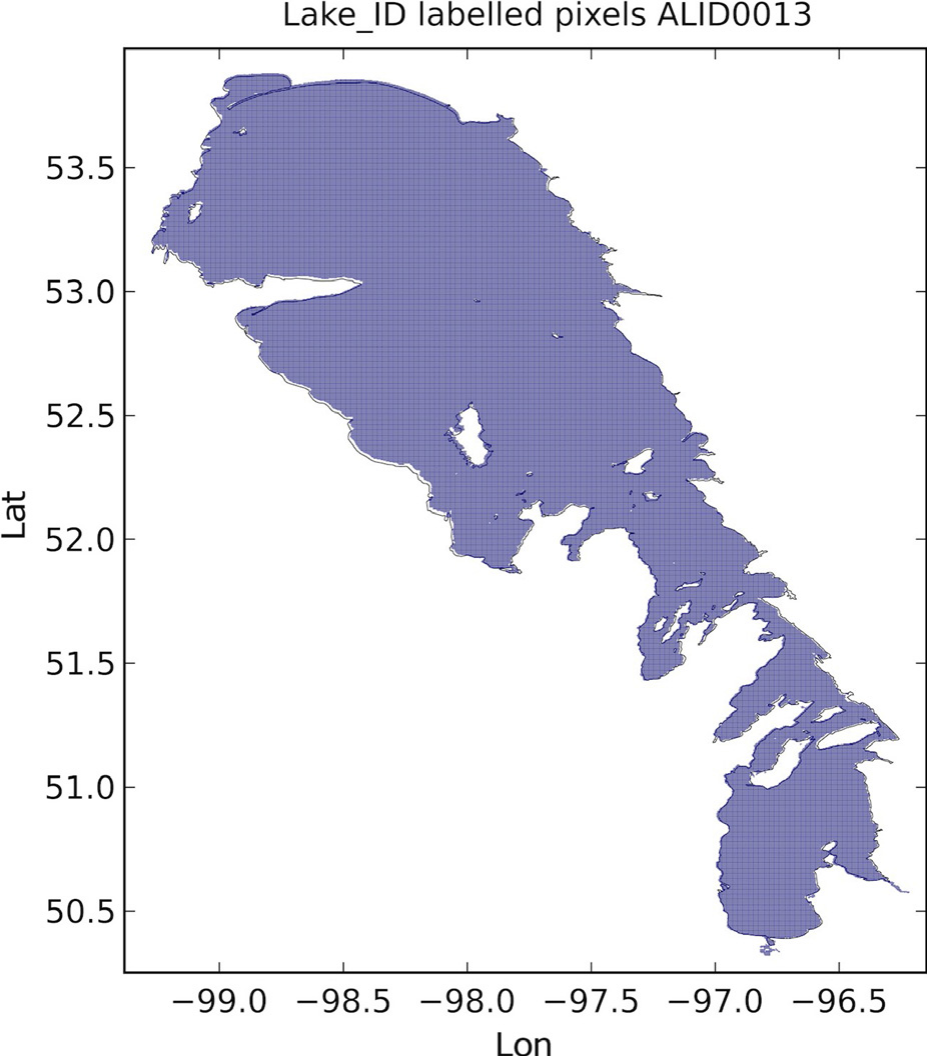
LC CCI cells labeled as Lake Winnipeg, and the corresponding GLWD polygon for ID **13**.

Figure 9 shows the pixels of the map shown in Figure 2 labeled as **13** for Lake Winnipeg in Canada.

Checking the dataset attributions has been performed mainly by visually comparing Landsat images from 2015, accessed via Google Earth, superimposing the GLDW polygons and the labeled water cells. We found this generally allowed an unambiguous interpretation of discrepancies between GLWD and our automated attribution (which was then sometimes refined by specific interventions). Inevitably, some judgements have been made, and users should be aware of that complications can arise for a particular case similar to those discussed below.

For the generation of identifiers dataset presented, the GLWD lake IDs have been utilized as the class values. The GLWD polygons were used as a starting point for the classification. Often a mismatch between the polygons and the extension of the water body in the LC CCI data was found. These arose partly due to the different observational periods, partly due to different resolutions and observational methods, and in some cases to obvious approximations in GLWD polygons. GLWD is from 2004. The static water‐bodies mask is the largest extent of the water bodies in the 2005 and 2010 period (and occasionally up to 2012 to avoid data voids). Since freshwater systems evolve because of natural events and human intervention, some mismatch is expected. Change has been especially rapid from the beginning of the 20th century (Lehner & Döll, 2004; UNESCO, 2009), and is very marked in the case of lakes like Lake Chad or the Aral Sea.

**Figure 10:**
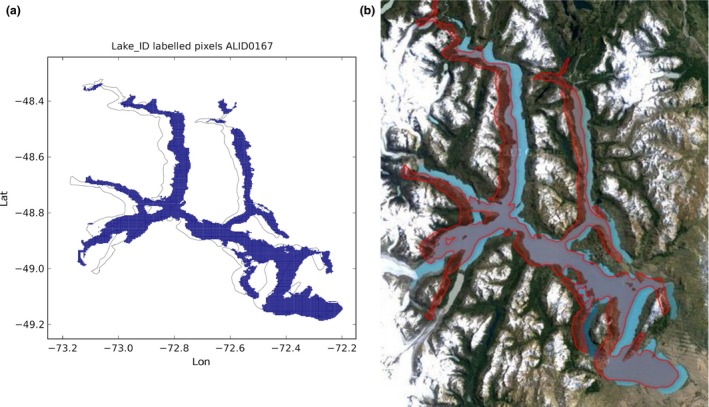
Lake San Martin in Chile/Argentina: the GLWD polygon shown together with the labeled pixels (a) and with the Landsat image from 2015, accessed through Google Earth ©2015 TerraMetrics (b).

An example of a mismatch between the polygon and the extension of the water body in the LC CCI mask is shown in Figure 10. The lake is San Martin in Chile/Argentina, and is among the largest 200 lakes globally. The comparison with the Landsat image from 2015, accessed through Google Earth, shown in Figure 10 indicates that the LC CCI water cells we have attributed to this lake offer a good representation of its location and extent, despite the discrepancy relative to the GLWD polygons. The source of the discrepancy does not appear to be due to differences in geographic coordinate systems used, since both LC CCI and the source dataset for the GLWD polygon in this case use the same datum (World Geodetic System 1984).

**Figure 11:**
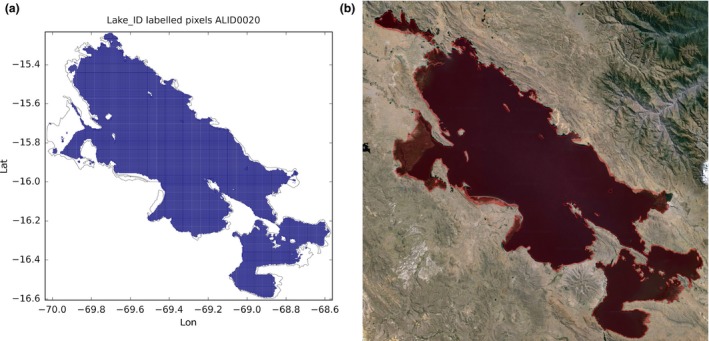
Lake Titicaca in Peru/Bolivia: the GLWD polygon shown together with the labeled pixels (a) and with the Landsat image from 2015, accessed through Google Earth ©2015 TerraMetrics (b). Note that land can be seen through the red overlay colour filling the GLWD polygon.

**Figure 12:**
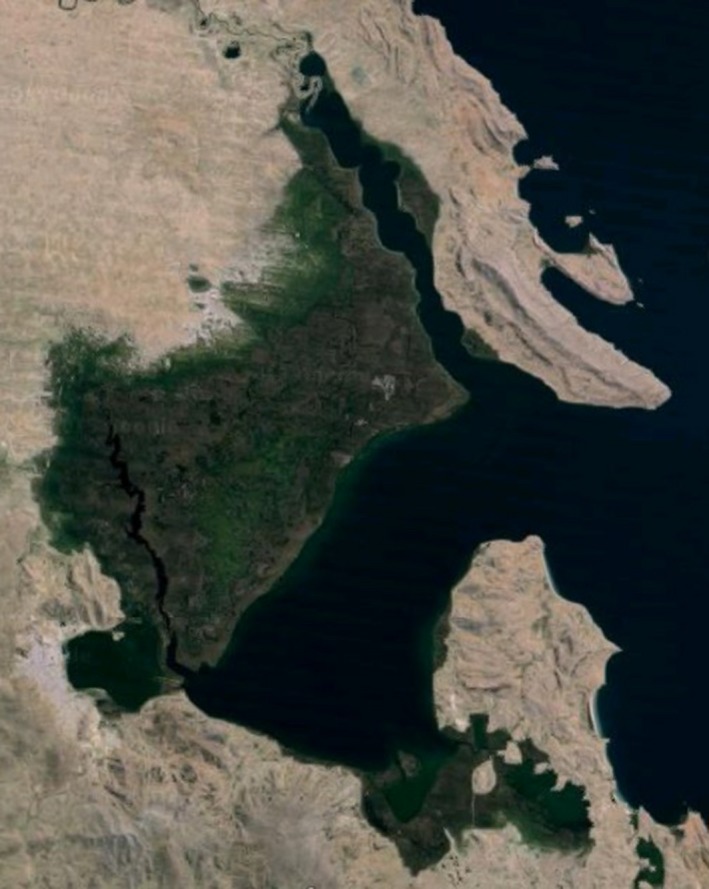
Lake Titicaca in Peru/Bolivia: a portion of the lake where the receding of the water level can be clearly seen. This fact is captured in the labeled pixels of the presented dataset. Landsat image from 2015, accessed through Google Earth ©2015 Landsat.

In Figure 11 Lake Titicaca is shown. In this case the mismatch is probably due to the fact that since 2000 Lake Titicaca has experienced constantly receding water levels (UNESCO, 2003) as can be seen in Figure 12. The labeled pixels of the presented dataset capture the receded lake.

**Figure 13:**
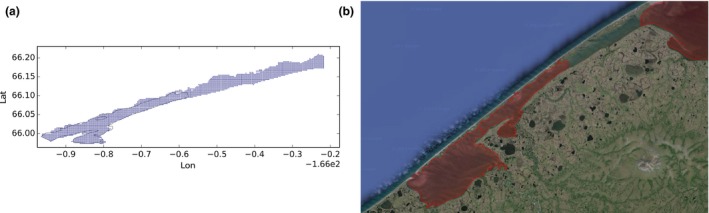
The Arctic lagoon in the United States: the GLWD polygon shown together with the labeled pixels (a) and with the Landsat image from 2015, accessed through Google Earth ©2015 TerraMetrics (b).

Sometimes the lakes in the dataset appear to be bigger than the GLWD polygons. One case is the Artic lagoon in the United States shown in Figure 13.

**Figure 14:**
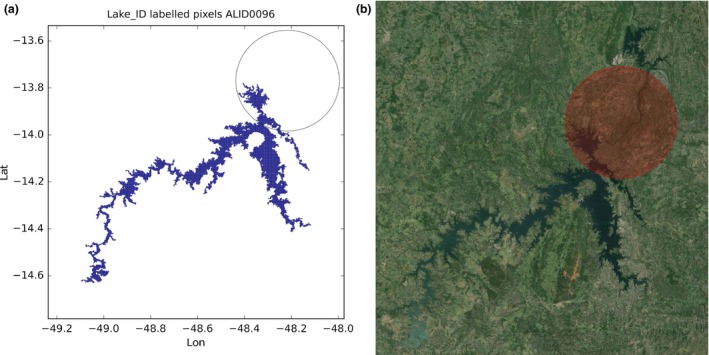
Lago de Serra da Mesa in Brazil: the GLWD polygon shown together with the labeled pixels (a) and with the Landsat image from 2015, accessed through Google Earth ©2015 TerraMetrics (b).

There are quite a few cases (especially in Brazil) in which the GLWD polygon is only a circle of size proportional to the amount of water stored in the water body. In this case, the full water body has been reconstructed. One example is Lago de Serra da Mesa in Brazil and it is shown in Figure 14.

**Figure 15:**
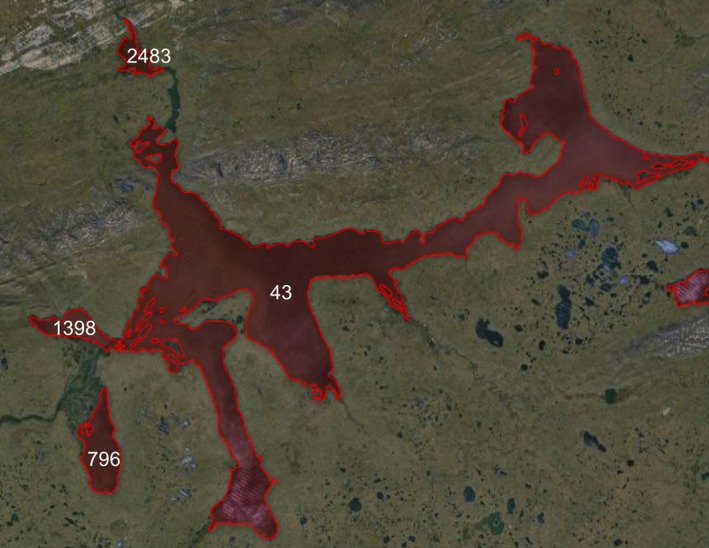
Lake Taymyr in Russia: different identifiers have been assigned to different portion of the lake. Landsat image from 2015, accessed through Google Earth ©2015 TerraMetrics.

**Figure 16:**
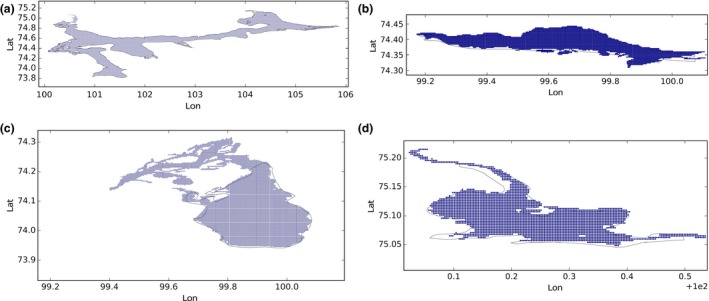
Lake Tymyr in Russia: the GLWD polygon shown together with the labeled pixels for the different portions of the lake. In (a) the pixels labeled with **43** are shown, in (b) with **1398**, in (c) with **796** and in (d) with **2483**.

In the GLWD database at times different portions of a water body that appear connected in LC CCI are labeled with different IDs. This is the case, for example, for Lake Taymyr in Russia where the lake has been split and assigned with four different labels as shown in Figure 15. Although apparently connected, the portions of the lake are quite distinct, and GLWD labeling has been maintained, generating four different GLWD‐lakes. Figure 16 shows the pixels labeled for the four different parts.

**Figure 17:**
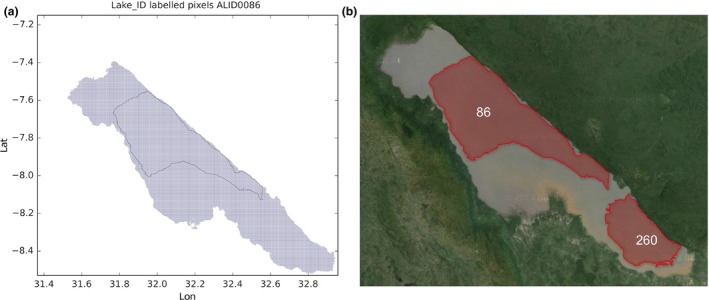
Lake Rukwa in Tanzania: the GLWD polygon shown together with the labeled pixels (a) and with the Landsat image from 2015, accessed through Google Earth ©2015 TerraMetrics (b).

There are other cases where the portions of a single water body are not distinct, yet are distinctly labeled in GLWD. An example is Lake Rukwa in Tanzania (see Figure 17). In this and similar cases, the judgement has been made as to which single label to attribute to the whole water body (in this case label **86**, subsuming GLWD ID **260**). We speculate that such cases arise where lake levels have increased significantly. The lakes where portions of a single water body have been labeled as one lake are reported below together with the IDs which have been eliminated. The eliminated IDs have been kept in the lake centre dataset described in the next section and have been assigned the name ‘X’ as to all the lakes that do not exist any longer.

**Figure 18:**
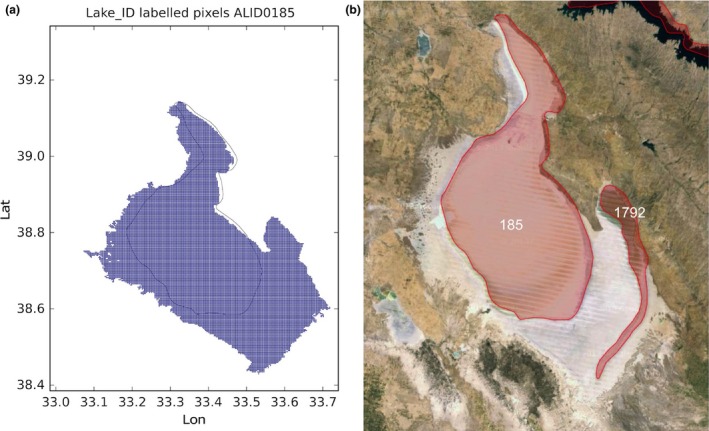
Lake Tuz in Turkey: the GLWD polygon shown together with the labeled pixels (a) and with the Landsat image from 2015, accessed through Google Earth ©2015 TerraMetrics (b).

**Figure 19:**
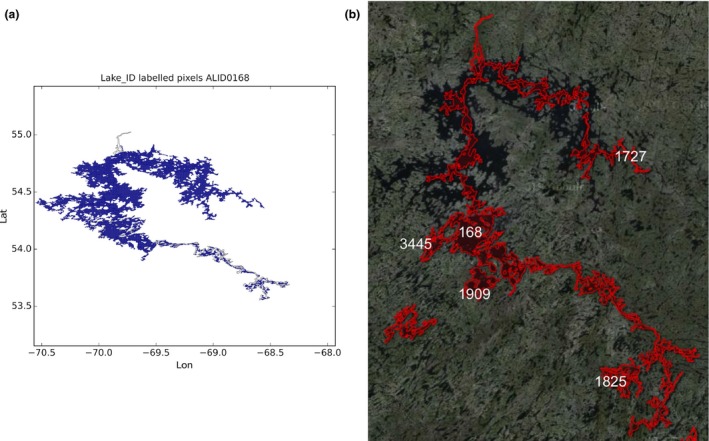
The Caniapiscau reservoir in Canada: the GLWD polygon shown together with the labeled pixels (a) and with the Landsat image from 2015, accessed through Google Earth ©2015 TerraMetrics (b).

**Figure 20:**
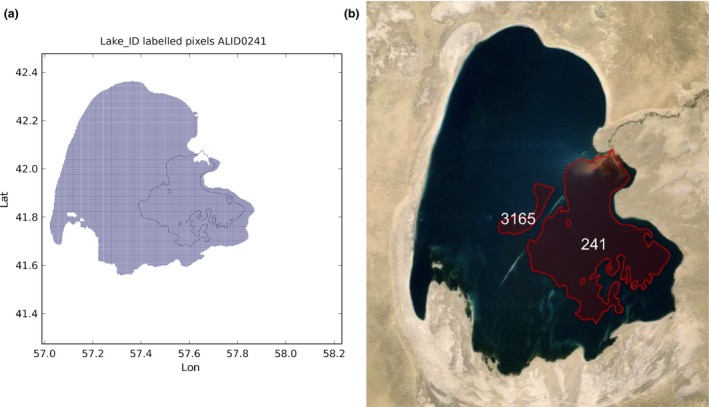
Lake Sarygamyş in Turkmenistan/Uzbekistan: the GLWD polygon shown together with the labeled pixels (a) and with the Landsat image from 2015, accessed through Google Earth ©2015 TerraMetrics (b).

- Lake Rukwa in Tanzania: the lake has been labeled with **86** and the label **260** has been assigned the name ‘X’ (see Figure 17)
- Lake Tuz in Turkey: the lake has been labeled with **185** and the label **1792** has been assigned the name ‘X’ (see Figure 18)
- Caniapiscau reservoir in Canada: the reservoir has been labeled with **168** and the label **1727** assigned the name ‘X’. The lakes **1909**,** 1825**, and **3445** have been kept separate (see Figure 19)
- Lake Sarygamyş in Turkmenistan/Uzbekistan: the lake has been labeled with **241** and the label **3165** has been assigned the name ‘X’ (see Figure 20).

**Figure 21:**
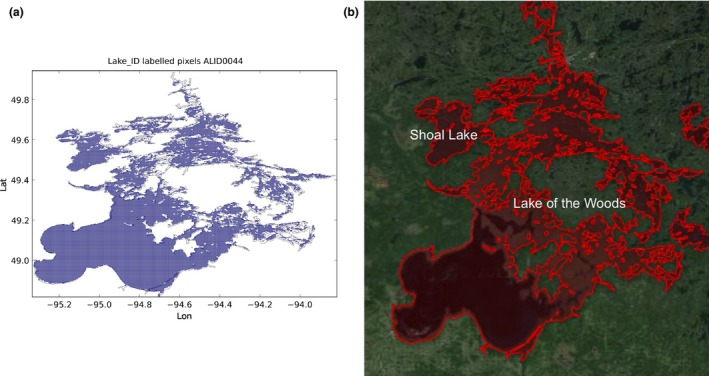
Lake of the Woods in Canada: the GLWD polygon shown together with the labeled pixels (a) and with the Landsat image from 2015, accessed through Google Earth ©2015 TerraMetrics (b).

There are cases where more than one water body has been assigned the same ID in GLWD. An example is Lake of the Woods shown in Figure 21 where also Lake Shoal and some extra water pixels have been included in the water body. In this case, the labeling has been maintained and for smaller lakes the names of the lakes have been reported.

There are other lakes in GLWD that do not seem to exist any longer, which gives rise to other ‘missing’ IDs. IDs for non‐existent and subsumed lakes are named with ‘X’ in the lake centre data described in the next section.

Regarding the sea and coastlines, the estuaries from the Global Estuary Database (Alder, 2003) have been used as a guideline to define estuarine waters as ‘sea’, with riverine waters as ‘other inland water’.

**Figure 22:**
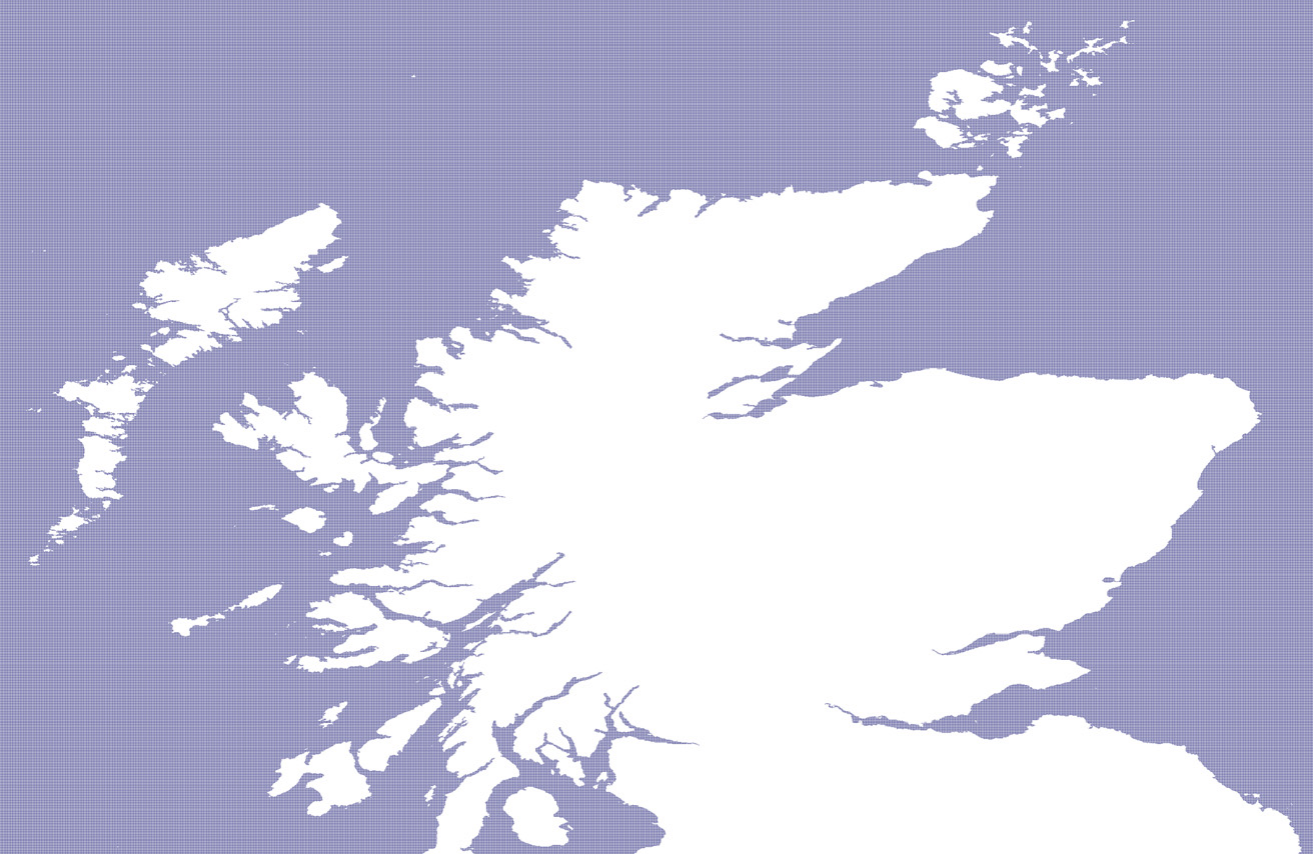
The sea around Scotland. The blue pixels are labeled as ocean, the white pixels are land or inland water.

An extract of the sea labeling is shown in Figure 22 where an area around Scotland is shown.

**Figure 23:**
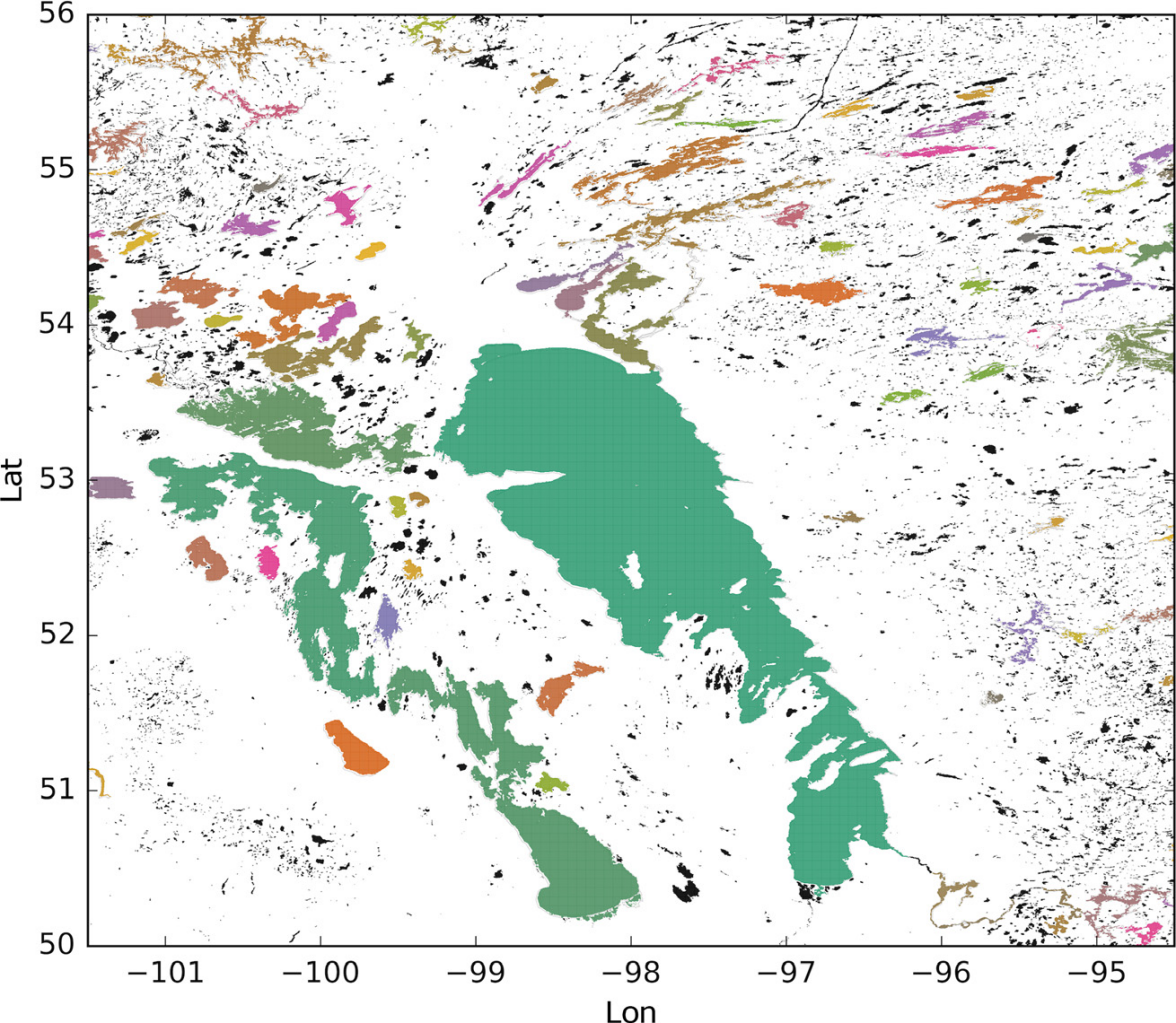
Extract of the water‐body IDs dataset around Lake Winnipeg in Canada. To each ID a unique color has been assigned. The color white corresponds to ‘land’ and the black color to ‘other inland water’. Each of the other colors corresponds to a specific classified lake.

**Figure 24:**
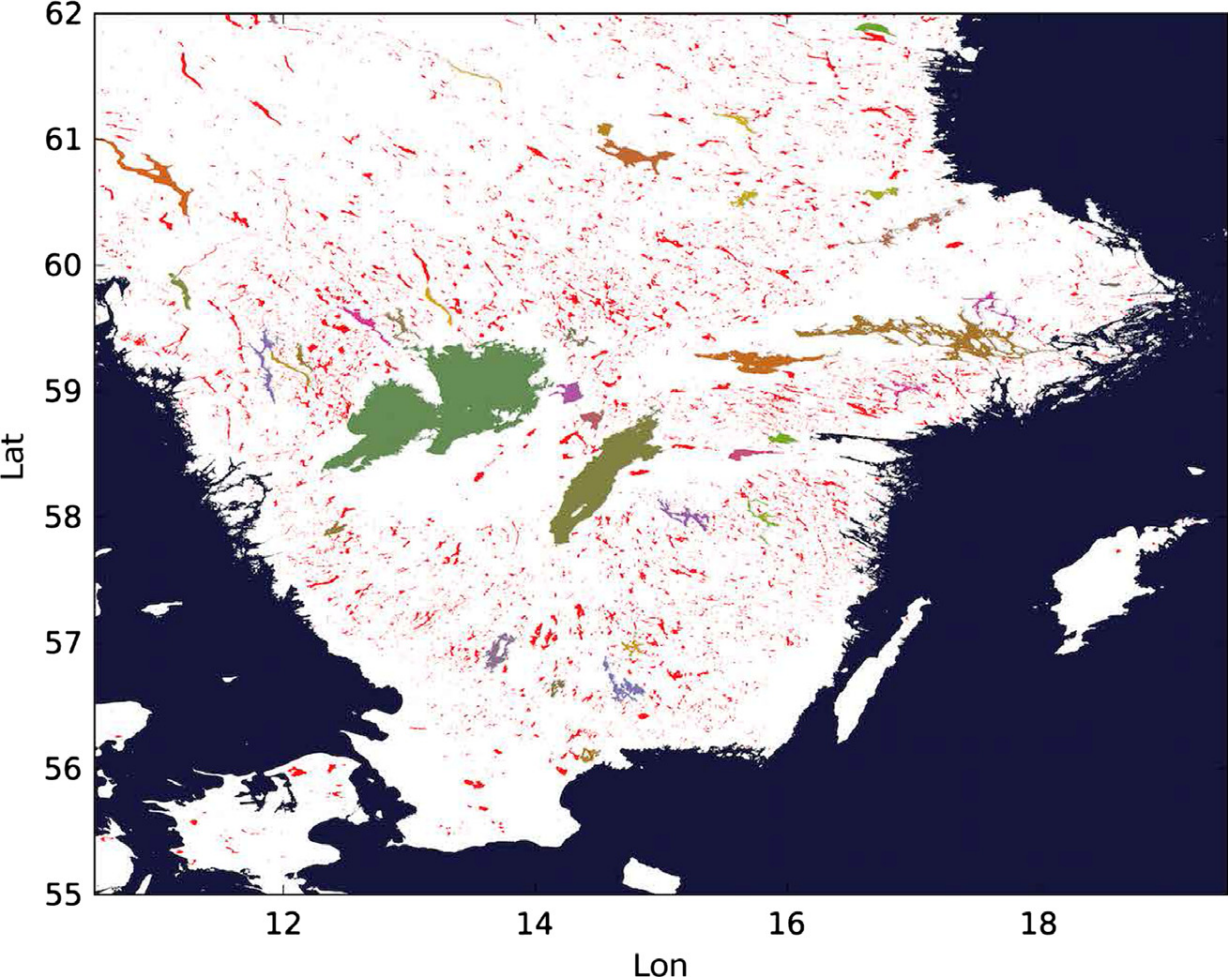
Extract of the water‐body IDs dataset in the south of Sweden. To each ID, a unique color has been assigned. The color white corresponds to ‘land’, the dark blue to ‘sea’ and the red to ‘other inland water’. Each of the other color corresponds to the ID of a specific classified lake.

An extract of the dataset around Lake Winnipeg in Canada is shown in Figure 23. Unclassified inland water is shown in black while classified lakes are shown in different colors. In Figure 24 water‐bodies labeled pixels are drawn for the south of Sweden. The dark blue pixels have been labeled as sea.

## 5 The lake‐centres dataset

We have also derived a dataset of lake ‘centres’. Various definitions of ‘centre’ could be used. We use the coordinates of the water cell which has the greatest distance to land. This means that the “centre" is always over water, which is not the case for some other potential definitions. From the point of view of remote sensing applications, this definition is useful for assessing whether a lake is a viable target.

The lake centres dataset has been derived from the distance‐to‐land dataset in conjunction with the water‐body identifiers dataset, for all the water bodies except ‘sea’ and ‘other water’. It is in a form of a list (a comma separated value file) containing the following information about the lakes that have been labeled with a GLWD identifier:

- GLWD identifier
- name
- country
- latitude/longitude coordinates of the centre
- the latitude/longitude coordinates of the corners of a box (orientated north‐south, east‐west) bounding the cells labeled as belonging to the specific lake.

The lakes that do not exist any longer have been maintained in the dataset and have been named with ‘X’. The lakes that do not seem to have a name have been assigned the name ‘Zzzz’.

## 6 Discussions and conclusions

Four consistent global datasets have been presented in this work: distance‐to‐land, distance‐to‐water, water‐body identifiers and water‐body centres (the latter two provided for the 3750 largest water bodies). During the generation and validation of the datasets some limitations of the GLWD database and of the LC CCI water‐bodies mask have been found. Regarding the GLWD dataset, for some lakes (like Lake San Martin) a mismatch between the GLWD polygon and the extension of the LC CCI mask has been found. In some cases, the mismatch can not necessarily be attributed to a real variation in areal coverage of the lake since the discrepancy takes the form of a shift. In cases investigated in detail, the shift does not appear to be attributable to inconsistent coordinate systems. It is beyond the scope of our work to explain the source of every mismatch. Comparison with Landsat images from 2015, accessed via Google Earth, shows greater consistency with the LC CCI mask than with the GLWD polygons. In other cases, the mismatch is plausibly due to a temporal variation in extension of the lake such is the case for Lake Titicaca, since the mapping behind the GLWD database dates from the early 1990s (various sources), while the LC CCI is covering the 2005‐2010 period. Also, in the GLWD database in some cases different portion of a single water body has been assigned with different IDs or different water bodies has been grouped within the same IDs and there are cases in which the water body does not seem to exist any longer.

During the validation of the distance‐to‐land dataset, some small islands (larger than the LC CCI resolution) have been found to be missing, e.g., most of the South Sandwich Islands and the Motu Nui island. At high latitudes, some water bodies are represented only at fairly coarse real resolution.

The derived datasets presented here are now in use in connection with thermal and microwave remote sensing applications, and may have utility for a wider cohort of users of water‐body information.
